# Allgemeinmedizinische Behandlungsfälle in einer universitären Notaufnahme vor und nach Einführung eines strukturierten Ersteinschätzungssystems

**DOI:** 10.1007/s00063-022-00950-4

**Published:** 2022-09-07

**Authors:** Tanja Schleef, Kristine Engeleit, Olaf Krause, Nils Schneider

**Affiliations:** 1 Institut für Allgemeinmedizin und Palliativmedizin, Medizinische Hochschule Hannover, Carl-Neuberg-Str. 1, 30625 Hannover, Deutschland; 2 Zentrum für Medizin im Alter, DIAKOVERE Henriettenstift, Hannover, Deutschland

**Keywords:** Notfallversorgung, Ambulante Versorgung, Emergency Severity Index, Niedrige Behandlungsdringlichkeit, Versorgungsforschung, Emergency service, hospital, Outpatient care, Emergency Severity Index, Non-urgent care, Health services research

## Abstract

**Hintergrund:**

In einer universitären Notaufnahme werden Patienten mit niedriger Behandlungsdringlichkeit und allgemeinmedizinisch-internistischen Beschwerden werktags durch Allgemeinärzte versorgt. Zur Festlegung der Behandlungsdringlichkeit wurde der Emergency Severity Index (ESI) eingeführt.

**Ziel der Arbeit:**

Ziel war es, die Auswirkung der ESI-Einführung auf die Zusammensetzung des allgemeinmedizinischen Patientenkollektivs zu untersuchen sowie die Verteilung der ESI-Kategorien bei diesen Patienten darzustellen.

**Methodik:**

Vergleich der allgemeinmedizinisch versorgten Patienten je 6 Monate vor (t0) und nach (t1) ESI-Einführung basierend auf Routinedaten und einem vom Allgemeinarzt auszufüllenden Erhebungsbogen. Die Analyse erfolgte deskriptiv und mittels χ^2^-Test bzw. t‑Test.

**Ergebnisse:**

Es wurden 615 Behandlungsfälle in t0 und 751 Fälle in t1 ausgewertet. Dabei zeigten sich keine signifikanten Unterschiede hinsichtlich des Alters, des Geschlechts, des Anteils der mit ärztlicher Einweisung vorstelligen Patienten oder der stationären Aufnahmen. Die ESI-Einstufung erfolgte überwiegend in die niedrigen Dringlichkeitskategorien ESI 5 (37 %) und ESI 4 (46 %), bei 8 % der Patienten in ESI 3 bzw. 2. Der prognostizierte Ressourcenbedarf stimmte für 76 % der Patienten in ESI 5, für 36 % in ESI 4 und für 44 % der Patienten in ESI 3 überein. Für 3 % der ESI-5-Patienten und 7 % der ESI-4-Patienten war eine stationäre Aufnahme erforderlich.

**Diskussion:**

Auch bei niedriger Behandlungsdringlichkeit kann eine stationäre Aufnahme indiziert sein, zudem weisen Abweichungen vom prognostizierten Ressourcenbedarf auf Triagierungsprobleme im untersuchten Patientenkollektiv hin. Zur Steuerung von Patienten in Versorgungsstrukturen außerhalb der Notaufnahme scheint die ESI-Ersteinschätzung nicht geeignet.

## Abstract (en)

**Background:**

In a university emergency department (ED), patients with low treatment urgency and general medical complaints are assigned to general practitioners within the ED. The Emergency Severity Index (ESI) was implemented to determine the urgency of treatment.

**Objectives:**

To investigate the impact of the ESI implementation on the patient population treated by general practitioners and to show the distribution of ESI categories among these patients.

**Methods:**

Comparison of emergency patients treated by general practitioners over 6 months before (t0) and after (t1) ESI implementation using routine data and an evaluation form to be completed by the general practitioner. The analysis was carried out descriptively and using the Χ^2^ test and t‑test.

**Results:**

At t0, 615 treatment cases and at t1 751 cases were analyzed. There were no significant differences in age, gender, the proportion of patients being referred to ED, or hospital admissions. The ESI classification was predominantly in the low urgency categories ESI 5 (37%) and ESI 4 (46%), with 8% of patients in ESI 3 or 2. The predicted resource needs matched for 76% of patients in ESI 5, for 36% in ESI 4, and for 44% of patients in ESI 3. Hospital admission was required for 3% of ESI 5 patients and 7% of ESI 4 patients.

**Conclusion:**

Even for patients with low treatment urgency, hospital admission may be indicated. In addition, differences from the predicted resource requirements indicate triaging problems in the patient population studied. Thus, the ESI assessment does not seem suitable to redirect patients to non-ED-based outpatient care.

An der Notfallversorgung sind zahlreiche Institutionen beteiligt. Sie umfasst die Versorgung ambulanter Notfälle durch Vertragsärzte bzw. durch den ärztlichen Bereitschaftsdienst außerhalb der üblichen Sprechstundenzeiten, das Rettungswesen und die Notaufnahmen der Krankenhäuser. Da der Zugang zum System der Notfallversorgung keiner Beschränkung unterliegt, können Patienten im subjektiven Notfall nach eigenem Ermessen entscheiden, welchen der 3 beteiligten Bereiche sie in Anspruch nehmen.

Durch die Beteiligung verschiedener Institutionen kann im Rahmen der Notfallversorgung ein umfassendes Spektrum von Versorgungsbedarfen abgedeckt werden, das von minderschweren gesundheitlichen Problemen bis hin zu akut lebensbedrohlichen Zuständen reicht [1, 15]. Während die Notaufnahmen der Krankenhäuser primär den Patienten in medizinisch kritischen Situationen und Indikation zur stationären Aufnahme vorbehalten sein sollen, liegt die Zuständigkeit für die ambulante Notfallversorgung in nicht lebensbedrohlichen Fällen bei den niedergelassenen Vertragsärzten bzw. bei dem über die Kassenärztlichen Vereinigungen organisierten ärztlichen Bereitschaftsdienst [1, 15, 23].

Für Patienten besteht allerdings auch im mutmaßlich „leichten“ Notfall bzw. Erkrankungsfall keine Verpflichtung, vorrangig die niedergelassenen Vertragsärzte oder den ärztlichen Bereitschaftsdienst in Anspruch zu nehmen [15]. Dies hat zur Folge, dass zahlreiche Patienten von der ihnen gegebenen Wahlmöglichkeit Gebrauch machen und in Akutsituationen die Notaufnahmen der Krankenhäuser aufsuchen [15, 18, 20, 24]. Verschiedene Studien beschreiben eine zunehmende Umverteilung der ambulanten Notfälle aus dem vertragsärztlichen Bereich in die Notaufnahmen der Krankenhäuser [10, 18, 24, 28]. So stieg die Gesamtzahl ambulanter Notfälle zwischen 2009 und 2019 von 18,3 Mio. auf 19 Mio. Fälle und ist insbesondere auf die Entwicklung der Fallzahlen in den Notaufnahmen zurückzuführen, die sich im gleichen Zeitraum von 8,2 Mio. auf 10,4 Mio. Fälle erhöhten [26]. In der Konsequenz versorgen Notaufnahmen ein stetig wachsendes sowie sehr heterogenes Patientenkollektiv, von denen ein Teil aus medizinischer Sicht keiner Versorgung in einer Notaufnahme bedarf [1, 18, 20].

Die Medizinische Hochschule Hannover (MHH) hat als Reaktion auf diese Entwicklung Allgemeinärzte (Fachärzte für Allgemeinmedizin oder hausärztlich tätige Internisten) in ihre Zentrale Notaufnahme (ZNA) integriert. Diese sind als Klinikärzte angestellt, d. h. sie sind weder freiberuflich noch vertragsärztlich tätig [21]. Die allgemeinmedizinische Patientenversorgung in der ZNA erfolgt seit 2014 montags bis freitags, jeweils von 10.00 bis 18.00 Uhr, und zielt auf eine allgemeinärztlich orientierte Diagnostik und Erstversorgung von Patienten mit weniger dringlichen Beschwerden. Hierfür stehen den Allgemeinärzten die gleichen Ressourcen des Krankenhauses wie den anderen Fachdisziplinen der ZNA zur Verfügung.

Um den Schweregrad der Erkrankung von Notfallpatienten zuverlässig zu erfassen, eine Kategorisierung sowie Priorisierung vorzunehmen und den geeigneten Behandlungsort innerhalb der Notaufnahme festzulegen, werden Triagesysteme eingesetzt [16, 29]. Diese strukturierte Form der Ersteinschätzung ist gemäß der Regelungen des Gemeinsamen Bundesausschusses in allen an der Notfallversorgung teilnehmenden Krankenhausnotaufnahmen mittlerweile verpflichtend durchzuführen [3]. Die in Deutschland am weitesten verbreiteten Triagesysteme sind das Manchester Triage System (MTS) und der Emergency Severity Index (ESI) [6, 16], letzterer wurde aufgrund seines ressourcenorientierten Ansatzes für die Größe der ZNA am Studienstandort (> 60.000 Patienten/Jahr) als praktikabler erachtet und kommt seit Mitte 2019 auch an der MHH zum Einsatz.

Ziel dieser Arbeit ist es, die Auswirkungen der Einführung des ESI als strukturiertes Ersteinschätzungsinstrument auf die Zusammensetzung des allgemeinmedizinisch versorgten Patientenkollektivs am Beispiel der MHH zu untersuchen sowie die Verteilung der ESI-Kategorien in diesem Patientenkollektiv darzustellen.

## Methoden

### Studiendesign

Es wurde eine retrospektive Analyse der allgemeinmedizinischen Behandlungsfälle in der ZNA der MHH über je 6 Monate vor (t0: 09/2018 bis 02/2019) und nach (t1: 09/2019 bis 02/2020) ESI-Einführung durchgeführt, wobei Patienten mit terminierter Wiedervorstellung in der ZNA von der Auswertung ausgeschlossen wurden. Mit Beginn von t1 war ESI seit 4 Monaten in Anwendung.

Vor Implementierung des ESI erfolgte die Ersteinschätzung aller vorstelligen Patienten sowie deren Zuordnung zur Fachdisziplin Allgemeinmedizin durch klinisch erfahrene Pflegefachkräfte nichtformalisiert auf Basis der präsentierten Leitsymptome. Im Mai 2019 wurde am Studienstandort der ESI (Version 4; [5]) zur strukturierten Ersteinschätzung etabliert. Hierzu durchliefen alle Pflegefachkräfte der ZNA eine 2‑tägige Schulung, die u. a. anhand von Fallvignetten durchgeführt wurde. Der ESI ist ein 5‑stufiges Triagesystem mit der Besonderheit, dass nur für die beiden Stufen der höchsten Behandlungsdringlichkeit (ESI 1 und ESI 2) eine Zeitvorgabe bis zum Behandlungsbeginn festgelegt ist. Bei Patienten, die keine sofortige oder zeitnahe Behandlung benötigen (ESI 3, 4 oder 5), fließt neben der Dringlichkeit des Leitsymptoms auch die Anzahl an voraussichtlich benötigten Ressourcen, bis eine Entscheidung über stationäre Aufnahme oder Entlassung gefällt werden kann, in die Kategorisierung ein [8, 16]. Unter Ressourcen werden in diesem Zusammenhang alle diagnostischen und therapeutischen Maßnahmen verstanden, die über die Anamnese, körperliche Untersuchung sowie einfache Medikations- und Wundversorgungsmaßnahmen hinausgehen. Definitionsgemäß sind über die körperliche Untersuchung hinausgehend keine Ressourcen in der ESI-Kategorie 5, eine Ressource in Kategorie 4 und 2 oder mehr Ressourcen in der Kategorie 3 vorgesehen [8, 16]. Nach der Einführung der strukturierten Form der Ersteinschätzung durchliefen alle in der ZNA vorstelligen Patienten eine Erstsichtung mittels ESI, wobei nach ZNA-interner Festlegung diejenigen Patienten der Allgemeinmedizin zugewiesen werden sollten, die sich mit einer niedrigen Behandlungsdringlichkeit gemäß ESI und allgemeinmedizinisch-internistischen Beschwerden vorstellen.

### Datenerhebung und Analyse

Die Datenbasis der retrospektiven Analyse bildeten die ZNA-Routinedaten des Krankenhausinformationssystems ergänzt um einen durch den diensthabenden Allgemeinarzt auszufüllenden zusätzlichen Dokumentationsbogen, der im Rahmen der Begleitevaluation der allgemeinärztlichen Tätigkeit in der ZNA zu jedem Patienten mitgeführt wurde.

Die als Freitexte dokumentierten Beratungsanlässe wurden orientierend an der International Classification of Primary Care (ICPC‑2, [13]) kategorisiert. Für die statistische Auswertung wurde IBM SPSS Statistics 26 (IBM Corporation, Armonk, NY, USA) verwendet. Deskriptive Ergebnisse wurden anhand von Häufigkeiten, prozentualen Angaben, Mittelwert und Standardabweichung dargestellt, Gruppenunterschiede wurden mittels t‑Test für normalverteilte Variablen, χ^2^-Test nach Pearson für kategoriale Variablen bzw. Linear-trend-Test für ordinalskalierte kategoriale Variablen analysiert. Ein *p*-Wert < 0,05 galt als signifikant. Die Speicherung und Verarbeitung aller personenbezogenen Daten erfolgte pseudonym über die jeweilige Fallnummer der Patienten.

### Ethik

Die Auswertung war Bestandteil der Begleitevaluation zur Integration der Allgemeinmedizin in die ZNA der MHH, für die ein positives Ethikvotum der Ethikkommission der MHH (Nr. 2067-2013) vorlag.

## Ergebnisse

Insgesamt gingen 1366 allgemeinmedizinische Behandlungsfälle in die Auswertung ein, davon 615 Behandlungsfälle im Zeitraum t0 und 751 Fälle im Zeitraum t1.

### Patientenkollektiv

Das Patientenkollektiv vor und nach ESI-Einführung unterschied sich nicht in Hinblick auf die soziodemographischen Merkmale oder die Art der Vor- und Weiterversorgung (Tab. 1). In beiden Beobachtungszeiträumen dominierten die Beratungsanlässe Bauchschmerzen (t0: *n* = 81/13,2 %; t1:* n* = 118/15,7 %) und Rückenschmerzen (t0: *n* = 69/11,2 %; t1: *n* = 76/10,1 %), etwa ein Viertel der Patienten wurde mit einem dieser beiden Beratungsanlässe vorstellig.

**Table 1:** 

ESI-Einführung	Vor ESI (*n* = 615)		Nach ESI (*n* = 751)		*p*
		(%)		(%)	
*Geschlecht*					0,248
Weiblich	344	(55,9)	392	(52,2)	
Männlich	265	(43,1)	343	(45,7)	
Keine Angabe	6	(1,0)	16	(2,1)	
*Alter* (Jahre; SD)	45,6	(19,9)	45,2	(19,4)	0,754
*Alterskategorien*					0,903
≤ 19 Jahre	27	(4,4)	27	(3,6)	
20–29 Jahre	138	(22,4)	162	(21,6)	
30–39 Jahre	110	(17,9)	142	(18,9)	
40–49 Jahre	88	(14,3)	118	(15,7)	
50–59 Jahre	88	(14,3)	105	(14,0)	
60–69 Jahre	52	(8,5)	75	(10,0)	
70–79 Jahre	47	(7,6)	46	(6,1)	
80–89 Jahre	34	(5,5)	45	(6,0)	
≥ 90 Jahre	12	(2,0)	8	(1,1)	
Keine Angabe	19	(3,1)	23	(3,1)	
*Art der Einweisung*					0,168
Selbstzuweisung^a^	490	(79,7)	495	(65,9)	
Ärztliche Einweisung	100	(16,3)	124	(16,5)	
Keine Angabe	25	(4,1)	132	(17,6)	
*Weiterversorgung*					0,990
Ambulant	524	(85,2)	648	(86,3)	
Stationäre Aufnahme	39	(6,3)	45	(6,0)	
Zuweisung innerhalb der ZNA^b^	26	(4,2)	33	(4,4)	
Sonstige^c^	11	(1,8)	13	(1,7)	
Keine Angabe	15	(2,4)	12	(1,6)	

^a^Da im Zugang zur ZNA nur zwischen Patienten ohne und mit ärztlicher Einweisung differenziert wird, können sich unter den Selbstzuweisungen auch Patienten befinden, die die ZNA über den Rettungsdienst erreicht haben

^b^Patienten wurden initial der Allgemeinmedizin zugeordnet, jedoch im Versorgungsverlauf an andere Fachdisziplinen innerhalb der ZNA abgegeben, sodass der weitere Verbleib unklar ist

^c^Gegangen ohne Arztkontakt/gegangen gegen ärztlichen Rat/Entlassung mit Termin zur Wiedervorstellung

### ESI-Einstufung und Ressourcenbedarf

Die Einstufung mittels ESI erfolgte überwiegend in die niedrigen Dringlichkeitskategorien 5 (*n* = 280/37,3 %) und 4 (*n* = 347/46,2 %) bei insgesamt 61 Patienten in ESI 3 bzw. 2 (8,1 %; Tab. 2).

**Table 2:** 

ESI-Einführung	Vor ESI (*n* = 615)		Nach ESI (*n* = 751)		*p*
	*n*	(%)	*n*	(%)	
*Ressourcenbedarf*					0,986
0	332	(54,0)	431	(57,4)	
1	204	(33,2)	211	(28,1)	
≥ 2	71	(11,5)	108	(14,4)	
Fehlend	8	(1,3)	1	(0,1)	
*Art der Ressource*					
Blutuntersuchung	215	(35,0)	254	(33,8)	0,550
EKG	80	(13,0)	104	(13,8)	0,713
Sonographie	15	(2,4)	38	(5,1)	0,014
Röntgen	21	(3,4)	34	(4,5)	0,319
CT/MRT	9	(1,5)	5	(0,7)	0,139
Fachärztliches Konsil	31	(5,0)	8	(1,1)	< 0,001
*ESI-Einstufung*					–
1	–	–	0	(0,0)	
2	–	–	4	(0,5)	
3	–	–	57	(7,6)	
4	–	–	347	(46,2)	
5	–	–	280	(37,3)	
Keine Einstufung erfolgt	–	–	18	(2,4)	
Keine Angabe	615	(100,0)	45	(6,0)	

*EKG* Elektrokardiographie, *CT* Computertomographie, *MRT* Magnetresonanztomographie

Für mehr als die Hälfte der Patienten im jeweiligen Beobachtungszeitraum (t0: *n* = 332/54,0 %; t1: *n* = 431/57,4 %) wurden über die körperliche Untersuchung hinaus keine Ressourcen benötigt, um eine Entscheidung über den weiteren Verbleib der Patienten zu treffen (Tab. 2). Für 275 Patienten (44,7 %) in t0 und 319 Patienten (42,5 %) in t1 hingegen war mindestens eine Ressource bzw. weiterführende diagnostische Maßnahme erforderlich. Am häufigsten wurde hierbei eine Blutuntersuchung durchgeführt. Dies betraf in t0 215 Patienten (78,2 %) bzw. in t1 254 Patienten (79,6 %). Ein Elektrokardiogramm (EKG) war in beiden Betrachtungszeiträumen die zweithäufigste diagnostische Maßnahme. Es wurde in t0 bei 80 Patienten (29,1 %) bzw. in t1 bei 104 Patienten (32,6 %) veranlasst. Signifikante Veränderungen nach ESI-Einführung zeigten sich bei den durchgeführten Sonographien sowie in den angeforderten fachspezialistischen Konsilen für das allgemeinmedizinisch versorgte Patientenkollektiv: Die Anzahl an Sonographien stieg von 15 (2,4 %) auf 38 (5,1 %; *p* = 0,014), während die fachspezialistischen Konsile sich von 31 (5,0 %) auf 8 (1,1 %) im Vergleichszeitraum verringerten (*p* < 0,001). Bei den anderen Parametern lagen keine statistisch signifikanten Unterschiede vor (Tab. 2).

### Über- und Untertriagierung

**Figure 1:**
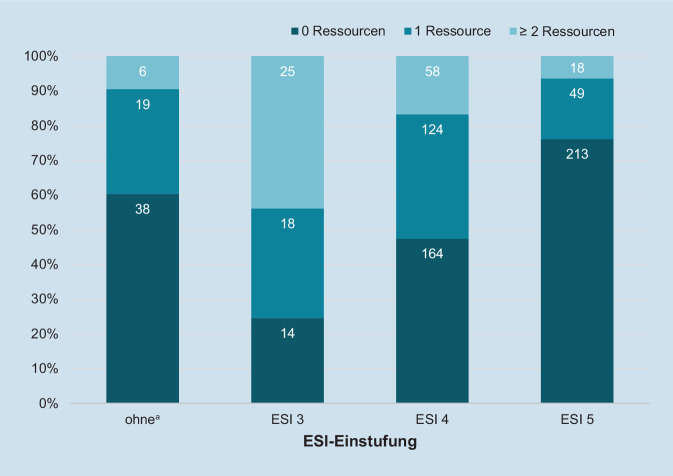

Der zum Zeitpunkt der Ersteinschätzung prognostizierte Ressourcenbedarf stimmte für 213 (76,1 %) von 280 Patienten in der ESI-Kategorie 5, für 124 (35,8 %) von 346 Patienten in ESI 4 und für 25 (43,9 %) von 57 Patienten der in ESI 3 eingestuften Patienten überein (Abb. 1). Entsprechend war insgesamt knapp die Hälfte (47,0 %) der in ESI 3, ESI 4 oder ESI 5 eingestuften Patienten gemessen an den tatsächlich benötigten Ressourcen über- bzw. untertriagiert, wobei es häufiger zu einer Übertriagierung (28,7 %) als zur einer Untertriagierung (18,3 %) kam. Richtig triagierte Patienten waren im Mittel 42,1 Jahre alt, hinsichtlich des Ressourcenbedarfs fehltriagierte Patienten waren mit durchschnittlich 48,9 Jahren signifikant älter (*p* < 0,001).

### Stationäre Aufnahme und ESI-Einstufung

Für 45 Patienten (6,0 %), darunter 18 Patienten (2,4 %) mit einer ärztlichen Einweisung, wurde in t1 eine stationäre Aufnahme veranlasst. Die stationären Aufnahmen aus den jeweiligen ESI-Kategorien lagen bei 9 Patienten (3,2 %) für ESI 5 und stiegen anteilig in den höheren Dringlichkeitskategorien auf 24 Patienten (6,9 %) für ESI 4 bzw. 9 Patienten (15,8 %) für ESI 3 an. Ein Patient wurde aus der ESI-Kategorie 2 stationär aufgenommen. Für 2 Patienten mit Indikation zur stationären Aufnahme lagen keine Angaben zur ESI-Einstufung vor.

## Diskussion

An der MHH wurde die Fachdisziplin Allgemeinmedizin in die ZNA intergiert, um diejenigen Patienten zu versorgen, die mit weniger dringlichen Gesundheitsproblemen aus dem allgemeinmedizinisch-internistischen Spektrum vorstellig werden. Die Einführung des ESI zeigte dabei keine Auswirkung auf die Zusammensetzung des allgemeinmedizinisch versorgten Patientenkollektivs. Lediglich bei den durchgeführten Sonographien sowie bei Konsilleistungen durch andere Fachdisziplinen der ZNA lagen signifikante Unterschiede zwischen den beiden Beobachtungszeiträumen vor, wobei die Anzahl an Sonographien in gleichem Umfang anstieg wie die angeforderten Konsile zurückgingen. Da andere diagnostische Maßnahmen, ebenso wie der Ressourcenbedarf in Summe, ohne signifikante Veränderungen blieben, ist der Unterschied bezüglich Sonographien und Konsilleistungen eher auf veränderte Kompetenzen wegen eines Personalwechsels innerhalb der in der ZNA tätigen Allgemeinärzte als auf eine Änderung der Zusammensetzung des Patientenkollektivs durch die ESI-Anwendung zurückzuführen. Erforderliche Sonographien wurden nach unserer Beobachtung vermehrt von den Allgemeinärzten selbst durchgeführt und somit entsprechend weniger als Konsilleistung bei anderen Fachdisziplinen angefordert.

Erwartungsgemäß ist die im Rahmen der Ersteinschätzung festgelegte Behandlungsdringlichkeit im dargestellten Patientenkollektiv insgesamt niedriger als in Gesamtkollektiven von Notaufnahmen: Während mehr als 80 % der zur Allgemeinmedizin triagierten Patienten auf die beiden niedrigsten ESI-Kategorien 4 und 5 entfielen, wurde im Aktin-Notaufnahme-Register – gespeist aus 15 deutschen Notaufnahmen – mit etwa 45 % ein deutlich kleinerer Anteil der Patienten in die beiden niedrigsten Dringlichkeitskategorien (MTS grün und blau bzw. ESI 4 und 5) eingestuft [2]. Entgegen der Erwartung gehören die der Allgemeinmedizin zugewiesenen Patienten jedoch nicht zwangsläufig der Kategorie mit der geringsten Behandlungsdringlichkeit an, die Mehrheit der Patienten wurde der ESI-Kategorie 4 zugeordnet.

Die Qualität der Ersteinschätzung bemisst sich daran, ob die festgelegte Dringlichkeitsstufe mit der tatsächlichen Behandlungsdringlichkeit übereinstimmt [4]. Da die wahre Behandlungsdringlichkeit nicht direkt gemessen werden kann, müssen Parameter, wie Mortalität, stationäre Aufnahmeraten oder Inanspruchnahme von Ressourcen, herangezogen werden, um die Qualität der Ersteinschätzung zu beurteilen [5, 16, 25]. Eine Orientierung an Mortalitätsraten ist in niedrigdringlichen Patientenkollektiven zur Bewertung der Triagierungsqualität nicht zielführend, allerdings weisen in unserer Untersuchung sowohl stationäre Aufnahmen aus niedrigen Triagekategorien als auch Abweichungen vom prognostizierten Ressourcenbedarf auf Triagierungsprobleme hin. Eine am tatsächlichen Ressourcenverbrauch gemessene Fehltriagierung betraf insgesamt 47 % der Patienten in den ESI-Kategorien 3, 4 und 5, wobei es häufiger zu einer Über- als zu einer Untertriagierung kam (28,7 % vs. 18,3 %). Dennoch wurde knapp ein Fünftel der allgemeinmedizinisch versorgten Patienten im Rahmen der Ersteinschätzung untertriagiert und benötigte mehr Ressourcen, als ursprünglich veranschlagt worden waren. Wesentliches Ziel der Ressourcenschätzung im Zuge der ESI-Anwendung ist es, komplexere – und damit ressourcenintensivere – Patientenfälle von denen mit einfacheren gesundheitlichen Problemen zu unterscheiden, wobei als komplexere Fälle diejenigen gelten, die 2 oder mehr Ressourcen benötigen [5]. Aus unseren Ergebnissen geht hervor, dass dies auf knapp 15 % der Patienten im Zeitraum t1 zutraf, allerdings wurden deutlich weniger Patienten bei Ersteinschätzung in die ESI-Kategorie 3 eingestuft. Der Ressourcenbedarf dieser Patienten wurde im Zuge der Ersteinschätzung nicht zuverlässig erkannt. Viele Faktoren können dabei eine Über- oder Untertriagierung bedingen. Da die triagierenden Pflegekräfte der universitären Notaufnahme mit einer Vielzahl schwerkranker und komplexer Fälle konfrontiert sind, kann es einerseits bei leichteren und weniger dringenden Fällen zu einer Übertriagierung kommen, weil möglichweise der Schweregrad der leichteren Fälle im Setting einer Universitätsklinik insgesamt überschätzt wird. Andererseits können bei vermeintlich trivialen Gesundheitsproblemen während der ärztlichen Anamnese zusätzliche Risikofaktoren oder Vorerkrankungen zutage treten und weiteren Ressourceneinsatz zur Abklärung nach sich ziehen, der zum Zeitpunkt der Ersteinschätzung zunächst nicht absehbar war.

Eine nichtdringliche Ersteinschätzung bedeutet nicht zwingend, dass eine inadäquate Vorstellung in der Notaufnahme vorliegt. Während in der Validierungsstudie der deutschsprachigen ESI-Version alle nach ESI 5 triagierten Patienten ambulant verblieben [9], konnte dieses Ergebnis in anderen Studien zu Ersteinschätzungssystemen nicht reproduziert werden. Bei 3–45 % der initial in die niedrigsten Dringlichkeitskategorien eingestuften Notfallpatienten war eine stationäre Aufnahme indiziert [12] und selbst bei isolierter Betrachtung von zunächst als nichtdringlich eingestuften Selbstzuweisern konnte eine Aufnahmequote von mehr als 10 % ermittelt werden [7]. Auch in unserem – überwiegend aus Selbstzuweisern bestehendem – Patientenkollektiv zeigte sich, dass mit einer Aufnahmerate von 3 % aus ESI 5 bzw. 7 % aus ESI 4 für einen kleinen, aber nicht unerheblichen Teil der Patienten eine stationäre Aufnahme erforderlich war.

In der Diskussion um die Anwendung von Ersteinschätzungsinstrumenten zeichnet sich – entgegen der ursprünglichen intendierten Identifikation von Risikopatienten sowie Priorisierung der Behandlungsdringlichkeit – zunehmend der Aspekt der Steuerung von Patienten in Versorgungsstrukturen außerhalb der Krankenhäuser ab [6, 17]. Studien bezüglich der Eignung des MTS zur Steuerung von Notfallpatienten ergaben, dass das MTS eher nicht geeignet ist, Patienten sicher in den niedergelassenen Bereich umzuleiten [7, 22]. Unsere Untersuchung zur Ersteinschätzung mittels ESI von weniger dringlichen Patienten mit allgemeinmedizinisch-internistischen Beratungsanlässen zeigte deutliche Triagierungsprobleme auf. Angesichts der dargestellten Ergebnisse scheint ein Zurückweisen niedrigdringlicher Patienten in Versorgungsstrukturen außerhalb der Notaufnahme alleine auf der Basis der ESI-Ersteinschätzung ebenfalls nicht gerechtfertigt.

Mit dem Ziel einer besseren Patientensteuerung wurde 2019 als weiteres Instrument zur Ersteinschätzung die im Auftrag des Zentralinstituts für die kassenärztliche Versorgung in Deutschland entwickelte „Strukturierte medizinische Ersteinschätzung in Deutschland“ (SmED) an mehreren Modellstandorten implementiert [27]. Nach einer Ersteinschätzung, die entweder telefonisch in den Vermittlungszentralen der teilnehmenden Kassenärztlichen Vereinigungen oder an gemeinsamen Standorten von Bereitschaftsdienstpraxen und Krankenhausnotaufnahmen erfolgt, wird der Patient entsprechend seiner Dringlichkeit der angemessene Versorgungsebene zugewiesen [11, 27]. Mit dem Einsatz im Rahmen der Telefontriage zielt SmED, im Gegensatz zu MTS und ESI, dabei auf eine Steuerung von Patienten vor dem Eintritt in das System der Notfallversorgung, während die Nutzung am „gemeinsamen Tresen“ im Prozess der Sichtung zur weiteren Behandlung ansetzt. Angesichts der Notaufnahme als Hochrisikobereich wird die Anwendung von SmED am gemeinsamen Tresen jedoch aus dem Blickwinkel der klinischen Notfallmedizin als kritisch erachtet [14].

Analysen der Notfallversorgung im europäischen Ausland beschreiben eine generelle Tendenz, die allgemeinärztliche Versorgung von Notfallpatienten mit niedrigem gesundheitlichem Risiko räumlich an die Krankenhäuser zu verlagern, diese jedoch weiterhin im Aufgabenbereich der Allgemeinärzte zu belassen [1, 19]. So ist beispielsweise in Dänemark für Patienten kein direkter Zugang zu einer Krankenhausnotaufnahme vorgesehen. Stattdessen ist im Rahmen der Patientensteuerung eine initiale telefonische Kontaktaufnahme mit einer Leitstelle erforderlich, die Notfallpatienten zu an Kliniken angesiedelten Notfallversorgungszentren leitet. Dort erfolgt eine abgestufte Notfallversorgung in Abhängigkeit vom gesundheitlichen Risiko sowie vermuteten Versorgungsbedarf entweder primärärztlich durch Allgemeinärzte oder notfallmedizinisch durch Krankenhausärzte [1, 19].

Bedingt durch das Fehlen effektiver Steuerungsmechanismen und die somit ungesteuerte, häufig nicht bedarfsgerechte Inanspruchnahme von Krankenhausnotaufnahmen steht das derzeitige deutsche System der Notfallversorgung vor großen Herausforderungen [1]. Die Integration von Allgemeinärzten in die Notaufnahme folgt somit der beobachteten Entwicklung im europäischem Ausland und ist eine Möglichkeit, für Patienten mit niedriger Behandlungsdringlichkeit eine allgemeinmedizinische Versorgung vorzuhalten, aber dennoch im Bedarfsfall auf diagnostische und therapeutische Ressourcen eines Krankenhauses zurückgreifen zu können.

### Limitationen

Bei der Einordnung der Ergebnisse und deren Übertragbarkeit sind folgende Limitationen zu berücksichtigen:

- Die Ergebnisse beziehen sich auf Patienten, die in einer universitären ZNA werktags zwischen 10.00 und 18.00 Uhr zur Fachrichtung Allgemeinmedizin triagiert wurden. Patienten, die sich nachts oder an Wochenenden in der ZNA vorstellten, wurden nicht in die Betrachtung einbezogen.
- Beim Zugang zur ZNA wurden Vorstellungen mit bzw. ohne ärztliche Einweisung unterschieden; ein Zugang über den Rettungsdienst wurde dabei nicht gesondert ausgewiesen.

## Fazit für die Praxis

- Allgemeinmedizinisch versorgte Notfallpatienten gehören nicht zwangsläufig den niedrigsten Dringlichkeitsstufen an.
- Auch bei niedriger Behandlungsdringlichkeit gemäß Ersteinschätzung kann ein stationärer Behandlungsbedarf vorliegen.
- Eine Steuerung nichtdringlicher Patienten an der Notaufnahme vorbei in andere ambulante Versorgungsstrukturen scheint auf der Basis des ESI nicht gerechtfertigt.
